# Identification of 2-keto-3-deoxy-d-Gluconate Kinase and 2-keto-3-deoxy-d-Phosphogluconate Aldolase in an Alginate-Assimilating Bacterium, *Flavobacterium* sp. Strain UMI-01

**DOI:** 10.3390/md15020037

**Published:** 2017-02-14

**Authors:** Ryuji Nishiyama, Akira Inoue, Takao Ojima

**Affiliations:** Laboratory of Marine Biotechnology and Microbiology, Faculty of Fisheries Sciences, Hokkaido University, Hakodate, Hokkaido 041-8611, Japan; nsym2480rj@eis.hokudai.ac.jp (R.N.); inouea21@fish.hokudai.ac.jp (A.I.)

**Keywords:** alginate degradation, 4-deoxy-l-erythro-5-hexoseulose uronic acid (DEH) metabolism, *Bacteroidetes*, *Proteobacteria*, *Flavobacterium*, 2-keto-3-deoxy-d-gluconate (KDG) kinase, 2-keto-3-deoxy-6-phosphogluconate (KDPG) aldolase, alginate-derived products

## Abstract

Recently, we identified an alginate-assimilating gene cluster in the genome of *Flavobacterium* sp. strain UMI-01, a member of *Bacteroidetes*. Alginate lyase genes and a 4-deoxy-l-erythro-5-hexoseulose uronic acid (DEH) reductase gene in the cluster have already been characterized; however, 2-keto-3-deoxy-d-gluconate (KDG) kinase and 2-keto-3-deoxy-6-phosphogluconate (KDPG) aldolase genes, i.e., *flkin* and *flald*, still remained uncharacterized. The amino acid sequences deduced from *flkin* and *flald* showed low identities with those of corresponding enzymes of *Saccharophagus degradans* 2-40^T^, a member of *Proteobacteria* (Kim et al., Process Biochem., 2016). This led us to consider that the DEH-assimilating enzymes of *Bacteroidetes* species are somewhat deviated from those of *Proteobacteria* species. Thus, in the present study, we first assessed the characteristics in the primary structures of KDG kinase and KDG aldolase of the strain UMI-01, and then investigated the enzymatic properties of recombinant enzymes, recFlKin and recFlAld, expressed by an *Escherichia coli* expression system. Multiple-sequence alignment among KDG kinases and KDG aldolases from several *Proteobacteria* and *Bacteroidetes* species indicated that the strain UMI-01 enzymes showed considerably low sequence identities (15%–25%) with the *Proteobacteria* enzymes, while they showed relatively high identities (47%–68%) with the *Bacteroidetes* enzymes. Phylogenetic analyses for these enzymes indicated the distant relationship between the *Proteobacteria* enzymes and the *Bacteroidetes* enzymes, i.e., they formed distinct clusters in the phylogenetic tree. recFlKin and recFlAld produced with the genes *flkin* and *flald,* respectively, were confirmed to show KDG kinase and KDPG aldolase activities. Namely, recFlKin produced 1.7 mM KDPG in a reaction mixture containing 2.5 mM KDG and 2.5 mM ATP in a 90-min reaction, while recFlAld produced 1.2 mM pyruvate in the reaction mixture containing 5 mM KDPG at the equilibrium state. An in vitro alginate-metabolizing system constructed from recFlKin, recFlAld, and previously reported alginate lyases and DEH reductase of the strain UMI-01 could convert alginate to pyruvate and glyceraldehyde-3-phosphate with an efficiency of 38%.

## 1. Introduction

Alginate is an acidic heteropolysaccharide comprising two kinds of uronic acid, β-d-mannuronate and α-l-guluronate [1,2,3]. This polysaccharide exists as a structural material in cell-wall matrices of brown algae and biofilms of certain bacteria. Since alginate solution shows high viscosity and forms an elastic gel upon chelating Ca^2+^, it has long been used as viscosifier and gelling agent in the fields of food and pharmaceutical industries. Alginate oligosaccharides produced by alginate lyases have also been recognized as functional materials since they exhibit various biological functions; e.g., promotion of root growth in higher plants [4,5], acceleration of growth rate of *Bifidobacterium* sp. [6], and promotion of penicillin production in *Penicillium chrysogenum* [7]. Anti-oxidant [8], anti-coagulant [9], anti-inflammation [10], and anti-infectious disease [11] are also bioactivities of alginate oligosaccharides. Recently, 4-deoxy-l-erythro-5-hexoseulose uronic acid (DEH), an end reaction product of alginate lyases, was proven to be available as a carbon source for ethanol fermentation by the genetically modified microbes [12,13,14]. Furthermore, 2-keto-3-deoxyaldonic acids like 2-keto-3-deoxy-d-gluconate (KDG) and 2-keto-3-deoxy-6-phosphogluconate (KDPG), which are intermediates in alginate metabolism, have been expected as leading compounds for antibiotics, antiviral agents, and other drugs and medicines [15]. Thus, such alginate-derived products are regarded as promising materials in various practical applications.

Alginate-degrading enzymes have been investigated in many organisms such as soil bacteria [16,17,18,19,20,21], marine bacteria [22,23,24,25,26,27,28,29], marine gastropods [30,31,32,33], and seaweeds [3,34]. Endolytic and exolytic alginate lyases split glycosyl linkages of alginate via β-elimination mechanism producing unsaturated oligosaccharides and monosaccharide, where a double bond is introduced between C4 and C5 of the newly formed non-reducing terminus [35]. Unsaturated monosaccharide, the end product of alginate lyases, is spontaneously [20] and/or enzymatically [36] converted to an open chain form, DEH, and further converted to KDG by the NAD(P)H-dependent DEH reductase. The KDG is phosphorylated to KDPG by KDG kinase and then split to pyruvate and glyceraldehyde-3-phosphate (GAP) by KDPG aldolase. The alginate-derived pyruvate and GAP are finally metabolized by Kreb’s cycle. Bacterial alginate lyases have been identified in many species, e.g., *Sphingomonas* sp. [16,17], *Flavobacterium* sp. [26,27], *Saccharophagus* sp. [22,23], *Vibrio* sp. [29], and *Pseudomonas* sp. [20,21]. *Sphingomonas* sp. strain A1 possesses four kinds of alginate lyases, A1-I–IV, whose sequential action completely depolymerizes alginate to DEH [16,17]. *Flavobacterium* sp. strain UMI-01 also possesses four kinds of alginate lyases, FlAlyA, FlAlyB, FlAlyC and FlAlex, whose cooperative action efficiently degrades alginate to DEH [27]. Meanwhile, *Saccharophagus degradans* strain 2-40^T^ possesses two kinds of alginate lyases, Alg7D and Alg17C, which degrade alginate to unsaturated disaccharide and DEH [22,23]. The alginate-derived DEH is reduced to KDG by NAD(P)H-dependent DEH reductases as described above. Recently, this enzyme was identified in *Sphingomonas* sp. strain A1 [18,19], *Flavobacterium* sp. strain UMI-01 [28], *S. degradans* strain 2-40^T^ [24], *Vibrio splendidus* 12B01 [13], and marine gastropod *Haliotis discus hannai* [37]. The bacterial DEH reductases were classified under short-chain dehydrogenases/reductases (SDR) superfamily, while the gastropod enzyme was identified as a member of the aldo-keto reductase (AKR) superfamily. Information about alginate lyases and DEH reductases has been continuously accumulated; however, KDG kinase and KDPG aldolase have not been so well investigated.

**Figure 1:**
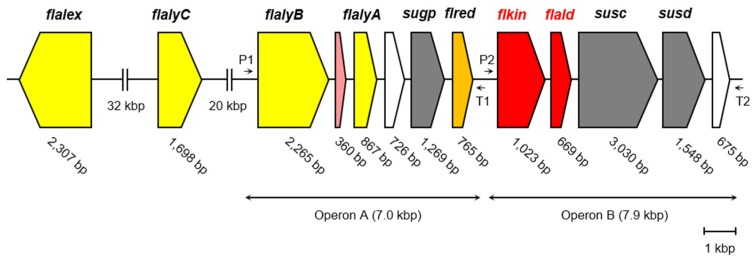
Alginate-assimilating enzyme genes in the genome of *Flavobacterium* sp. strain UMI-01. Yellow, alginate-lyase genes; pink, KdgF-like protein gene; white, transcriptional regulator-like protein genes; gray, membrane transporter-like genes; orange, 4-deoxy-l-erythro-5-hexoseulose uronic acid (DEH) reductase gene; red, 2-keto-3-deoxy-d-gluconate (KDG) kinase-like gene and 2-keto-3-deoxy-6-phosphogluconate (KDPG) aldolase-like gene. Arrows P1 and P2 and arrows T1 and T2 indicate predicted promoters and terminators, respectively.

Under these circumstances, DEH reductase, KDG kinase, and KDPG aldolase were recently characterized in *S. degradans* 2-40^T^, a member of the phylum *Proteobacteria* [25]. The combined action of these enzymes could convert DEH to pyruvate and GAP in vitro. On the other hand, we also found the existence of alginate-assimilating gene cluster in the genome of *Flavobacterium* sp. strain UMI-01, a member of the phylum *Bacteroidetes* [27,28]. The endolytic and exolytic alginate lyase genes, *flalyA* and *flalyB*, and a DEH reductase gene, *flred*, are located in operon A, and KDG kinase-like gene *flkin* (GenBank accession number, BAQ25538) and KDPG aldolase-like gene *flald* (GenBank accession number, BAQ25539) are in operon B (Figure 1). The alginate lyases and DEH reductase of this bacterium have already been characterized [26,27,28]; however, KDG kinase and KDPG aldolase have not been identified yet. The amino acid sequences deduced from *flkin* and *flald* showed only 19% and 22% identities, respectively, with those of the corresponding enzymes from *S. degradans* 2-40^T^ [25]. These low sequence identities suggest that the properties of *Flavobacterium* (*Bacteroidetes*) enzymes may be somewhat different from those of *Saccharophagus* (*Proteobacteria*) enzymes. Therefore, in the present study, we first characterized the primary structures of KDG kinase and KDPG aldolase, FlKin and FlAld, of the strain UMI-01 compared with those of other bacterial enzymes. Then, we investigated enzymatic properties of proteins encoded by *flkin* and *flald* using recombinant enzymes, recFlKin and recFlAld. Furthermore, we constructed an in vitro alginate-metabolizing system using recFlKin and recFlAld, along with recombinant alginate lyases and DEH reductase of this bacterium to confirm that this enzyme system can produce pyruvate and GAP from alginate in vitro.

## 2. Results

### 2.1. Characteristics in the Primary Structures of FlKin and FlAld

**Figure 2:**
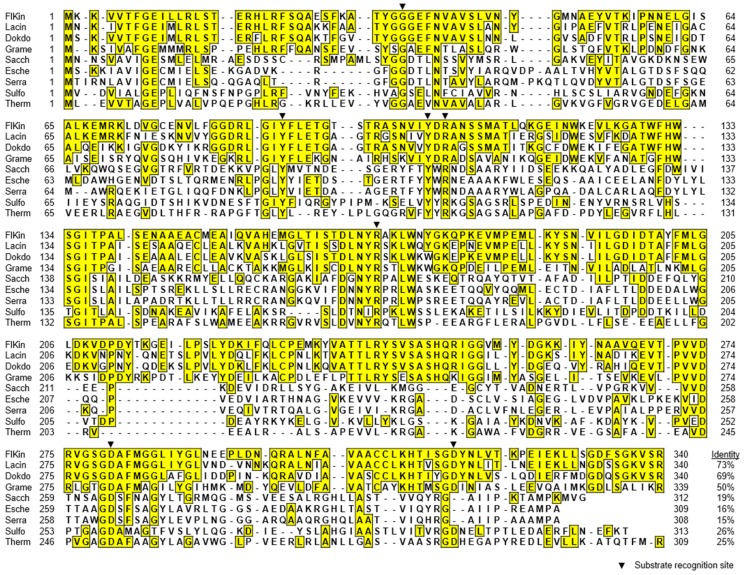
Multiple alignment for amino acid sequences of FlKin and other KDG kinases. Closed triangles indicate substrate-recognition residues of KDG kinase from *Sulfolobus solfataricus* [42]. FlKin, KDG kinase from *Flavobacterium* sp. strain UMI-01 (GenBank accession number, BAQ25538); Lacin, KDG kinase-like protein from *Lacinutrix* sp. 5H-3-7-4 (GenBank accession number, AEH01605); Dokdo, KDG kinase-like protein from *Dokdonia* sp. MED134 (GenBank accession number, WP_013749800); Grame, KDG kinase-like protein from *Gramella forsetii* KT0803 (GenBank accession number, CAL66135); Sacch, KDG kinase from *Saccharophagus degradans* 2-40^T^ (GenBank accession number, ABD82535) [25]; Esche, KDG kinase from *Escherichia coli* (GenBank accession number, WP_024175791) [38]; Serra, KDG kinase from *Serratia marcescens* (GenBank accession number, ABB04497) [39]; Sulfo, KDG kinase from *Sulfolobus solfataricus* (GenBank accession number, WP_009991690) [40,41,42]; Therm, KDG kinase from *Thermus thermophiles* (GenBank accession number, WP_011229211) [43].

**Figure 3:**
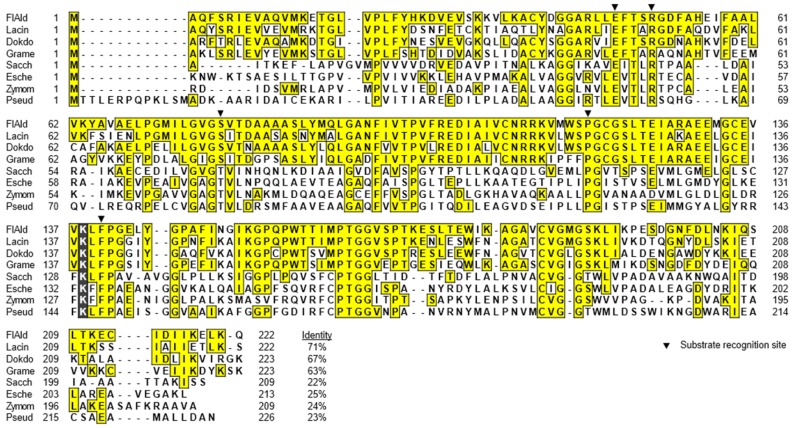
Multiple alignment for amino acid sequences of FlAld and other KDPG aldolases. Gray box and closed triangles indicate catalytic and substrate-recognition residues of KDPG aldolase from *E. coli* [44,45], respectively. FlAld, KDPG aldolase from *Flavobacterium* sp. strain UMI-01 (GenBank accession number, BAQ25539); Lacin, KDPG aldolase-like protein from *Lacinutrix* sp. 5H-3-7-4 (GenBank accession number, AEH01606); Dokdo, KDPG aldolase-like protein from *Dokdonia* sp. MED134 (GenBank accession number, WP_013749799); Grame, KDPG aldolase-like protein from *G. forsetii* KT0803 (GenBank accession number, CAL66136); Sacch, KDPG aldolase from *S. degradans* 2-40^T^ (GenBank accession number, ABD80644) [25]; Esche, KDPG aldolase from *E. coli* (GenBank accession number, WP_000800517) [44,45]; Zymom, KDPG aldolase from *Zymomonas mobilis* (GenBank accession number, S18559) [44]; Pseud, KDPG aldolase from *Pseudomonas putida* (GenBank accession number, WP_016501275) [44,46].

**Figure 4:**
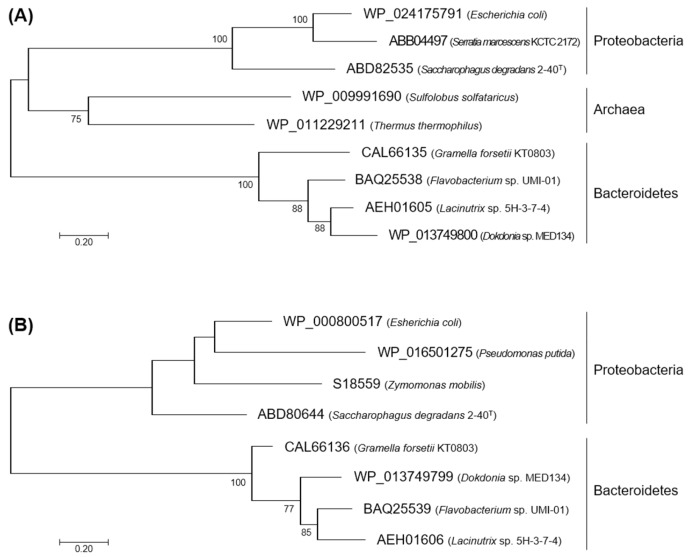
Phylogenetic trees for KDG kinases and KDPG aldolases. Phylogenetic analyses were carried out using amino acid sequences of KDG kinases from *Proteobacteria*, *Archaea* and *Bacteroidetes* species (**A**) and KDPG aldolases from *Proteobacteria* and *Bacteroidetes* species (**B**). Amino acid sequences of KDG kinases and KDPG aldolases were retrieved from the draft or complete genome data deposited in GenBank. Accession numbers for enzyme sequences along with the bacterial species are indicated in the right of each branch. Bootstrap values above 50% are indicated on the root of branches. Scale bar indicates 0.20 amino acid substitution.

Deduced amino acid sequences of *flkin* and *flald* were compared with those of KDG kinases and KDPG aldolases from several *Proteobacteria* and *Bacteroidetes* species. Enzymes from two *Archaea* species are also included in the comparison of KDG kinases. FlKin showed considerably low amino acid identity (15%–26%) with KDG kinases from *Proteobacteria* species, i.e., *Escherichia coli* (GenBank accession number, WP_024175791) [38], *Serratia marcescens* (GenBank accession number, ABB04497) [39], and *S. degradans* 2-40^T^ (GenBank accession number, ABD82535) [25], and archaea, i.e., *Sulfolobus solfataricus* (GenBank accession number, WP_009991690) [40,41,42] and *Thermus thermophiles* (GenBank accession number, WP_011229211) [43] (Figure 2). Meanwhile, the sequence of FlKin showed relatively high identities (47%–68%) with the enzymes from *Bacteroidetes* species, i.e., *Gramella forsetii* KT0803 (GenBank accession number, CAL66135), *Dokdonia* sp. MED134 (GenBank accession number, WP_016501275), and *Lacinutrix* sp. 5H-3-7-4 (GenBank accession number, AEH01605). However, substrate-recognition residues of KDG kinase, which were identified in the *S. solfataricus* enzymes [42], i.e., Gly34, Tyr90, Tyr106, Arg108, Arg166, Asp258, and Asp294, were entirely conserved in FlKin as Gly34, Tyr89, Tyr104, Arg106, Arg169, Asp280, and Asp317, respectively. FlAld also showed low amino acid identity (22%–25%) with KDPG aldolases from *Proteobacteria* species such as *E. coli* (GenBank accession number, WP_000800517) [44,45], *Zymomonas mobilis* (GenBank accession number, S18559) [44], *Pseudomonas putida* (GenBank accession number, WP_016501275) [44,46], and *S. degradans* 2-40^T^ (GenBank accession number, ABD80644) [25] (Figure 3). Meanwhile, the sequence identities between FlAld and enzymes from other *Bacteroidetes* species such as *G. forsetii* KT0803 (GenBank accession number, KT0803), *Dokdonia* sp. MED134 (GenBank accession number, WP_013749799), and *Lacinutrix* sp. 5H-3-7-4 (GenBank accession number, AEH01606) were 61%–65%. Catalytic residue Lys133 and substrate-recognition residues, Glu45, Arg49, Thr73, Pro94 and Phe135 identified in the *E. coli* enzyme [45], were conserved in FlAld except for the substitution of Thr73 by Ser. Phylogenetic analyses for KDG kinases and KDPG aldolases (Figure 4A,B) suggested that the *Bacteroidetes* enzymes are somewhat phylogenetically deviated from the *Proteobacteria* (and *Archaea*) enzymes. Therefore, we decided to examine if FlKin and FlAld of the strain UMI-01 actually possess KDG kinase and KDPG aldolase activities.

### 2.2. Production of recFlKin and recFlAld, and Their Reaction Products

**Figure 5:**
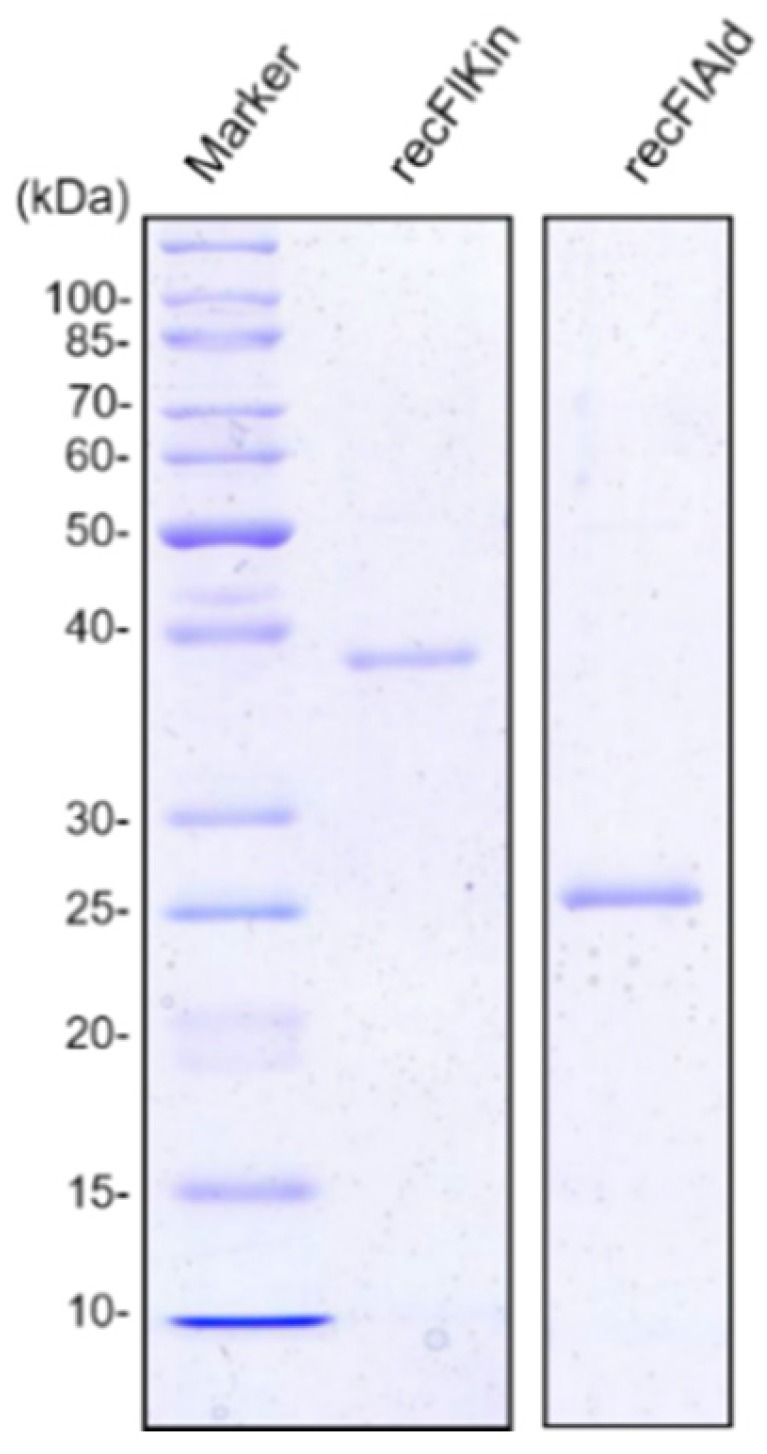
SDS-PAGE for recFlKin and recFlAld. Recombinant enzymes were purified Ni-NTA affinity chromatography and subjected to 0.1% SDS–10% polyacrylamide-gel electrophoresis. Proteins in the gel were stained by Coomassie Brilliant Blue R-250. Marker, molecular weight markers (Protein Ladder Broad Range, New England Biolabs, Ipswich, MA, USA).

Coding regions of *flkin* and *flald* were amplified by PCR with specific primers listed in Table 1, cloned into pCold vector and expressed in *E. coli* BL21 (DE3). The recombinant enzymes were purified by Ni-NTA affinity chromatography. Molecular masses of recFlKin and recFlAld estimated by SDS-PAGE were 39 kDa and 26 kDa, respectively (Figure 5). These values were consistent with the calculated molecular masses of these enzymes, i.e., 39,391 Da and 25,808 Da, which include 8 × Gly + 8 × His-tag [26].

**Figure 6:**
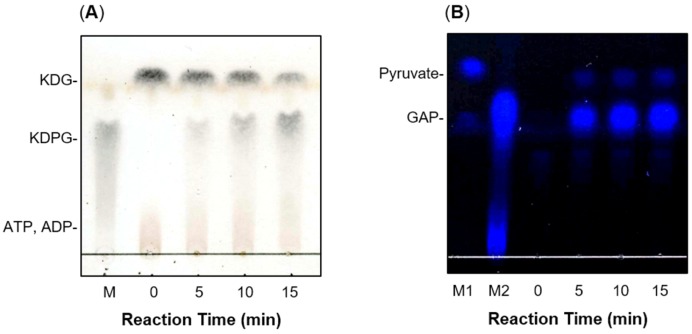
Thin-layer chromatography (TLC) analyses for reaction products of recFlKin and recFlAld. (**A**) Reaction products produced by recFlKin. The reaction products were visualized by spraying 10% (*v*/*v*) sulfuric acid in ethanol followed by heating at 130 °C for 10 min. M, standard KDPG; (**B**) Reaction products of recFlAld. The reaction products were visualized with 0.5% (*w*/*v*) 2,4-dinitrophenylhydrazine (DNP)–20% (*v*/*v*) sulfuric acid. The color was graphically inverted to ease the recognition of spots. M1, standard pyruvate; M2, standard glyceraldehyde-3-phosphate (GAP). Stained materials near the original position are GAP oligomers.

**Figure 7:**
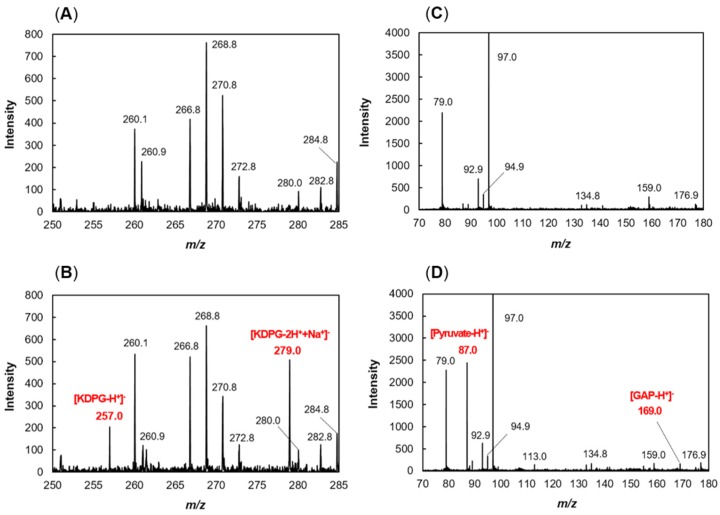
Mass spectrometry for reaction products of recFlKin and recFlAld. The reaction products prepared as in Section 4.10 were subjected to matrix-assisted laser desorption ionization-time of flight mass spectrometer (MALDI-TOF) mass spectrometry, and analyzed by negative-ion mode. (**A**,**B**) KDG before and after the recFlKin reaction, respectively; (**C**,**D**) KDPG before and after the recFlAld reaction, respectively. Reaction products are indicated with red letters along with molecular masses above the peaks.

The recFlKin was allowed to react with KDG in the presence of ATP. TLC analysis suggested that the reaction product was KDPG (Figure 6A). Then, the molecular mass of the reaction product was determined by matrix-assisted laser desorption ionization-time of flight mass spectrometer (MALDI-TOF) mass spectrometry (Figure 7A,B). The 257 *m*/*z* peak was considered to be that of KDPG (MW = 258), and the 279 *m*/*z* peak was considered to be that of a sodium-salt form of KDPG. These results indicate that the reaction product of recFlKin is KDPG. Thus, we concluded that the protein encoded by *flkin* is KDG kinase. Here, it should be noted that the peak intensities of KDPG were considerably low. This was ascribable to the low ionization level of KDPG. Therefore, we attempted to improve the signal intensity of KDPG using other matrices, e.g., 2,5-dihydroxybenzoic acid and α-cyano-4-hydroxycinnamic acid. Unfortunately, signal intensity of KDPG was not improved much. We still need to investigate the suitable conditions for the detection of KDPG.

Reaction products of recFlAld were also analyzed by TLC (Figure 6B). recFlAld produced two kinds of reaction products with different mobility on TLC. According to their mobility, they were regarded as pyruvate and GAP. The staining intensity of pyruvate was significantly low compared with that of GAP. This difference was ascribable to the difference in the reactivity between pyruvate and GAP with 2,4-dinitrophenylhydrazine (DNP). Namely, GAP showed much higher reactivity with DNP than pyruvate. Then, the reaction products of recFlAld were subjected to MALDI-TOF mass spectrometry. The 87 *m*/*z* and 169 *m*/*z* peaks corresponding to pyruvate (MW = 88) and GAP (MW = 170), respectively, were observed. The peak intensity of GAP was small (Figure 7C,D). This appeared to be due to the decomposition of GAP during the mass spectrometric analysis. Thus, we may conclude that recFlAld is the KDPG aldolase that splits KDPG to pyruvate and GAP.

### 2.3. Enzymatic Properties of recFlKin and recFlAld

We first investigated the kinetic parameter for recFlAld, since recFlAld was necessary for the KDG kinase assay. In the present study, the kinase activity was assayed by quantifying the pyruvate produced from KDPG by the action of recFlAld. KDPG-derived pyruvate was determined by the lactate dehydrogenase (LDH)–NADH system as described in Section 4.6. In the equilibrium state of recFlAld reaction, pyruvate concentration reached 1.2 mM. Since the KDPG concentration was originally 5 mM, that in the equilibrate state was regarded as 3.8 mM. From these values the equilibrium constant (*K*_eq_) and Δ*G*° were calculated to be 3.8 × 10^−1^ M and +0.57 kcal/mol, respectively. This indicated that the equilibrium position of KDPG–aldolase reaction is slightly shifted toward the KDPG side. Next, we determined the reaction rate of recFlAld by the LDH–NADH method. By this method, the specific activity of recFlAld was estimated to be 57 U/mg at pH 7.4 and 30 °C. Coexistence of LDH–NADH in the reaction mixture could extend the aldolase reaction longer time by decreasing pyruvate concentration in the reaction equilibrium.

Next, KDG kinase activity of recFlKin was determined by using recFlAld and LDH–NADH. recFlKin was allowed to react with KDG in the presence of ATP at 30 °C and the reaction was terminated by heating at 100 °C for 3 min at the reaction times 1, 15, and 30 min. The KDPG produced in the reaction mixture was then split to pyruvate and GAP by recFlAld, and the pyruvate was quantified by the LDH–NADH system. At reaction time 90 min, recFlKin was found to produce 1.7 mM KDPG from 2.5 mM KDG at ~70% efficiency with the specific activity 0.72 U/mg. recFlKin showed an optimal temperature and pH at around 50 °C and 7.0, respectively, and was stable at 40 °C for 30 min.

### 2.4. Construction of In Vitro Alginate-Metabolizing System Using Recombinant Enzymes

**Figure 8:**
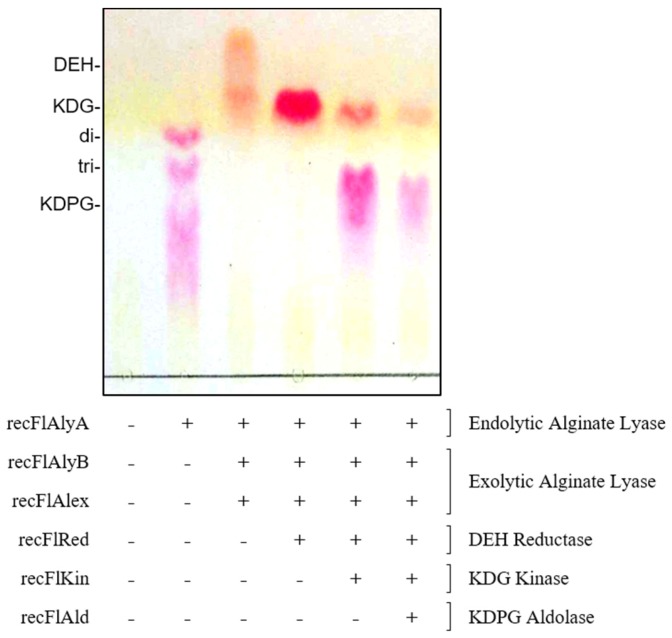
Construction of in vitro alginate-metabolizing system using recombinant enzymes. Alginate was allowed to react with recFlAlyA, recFlAlyB, recFlAlex, recFlRed, recFlKin, and recFlAld in various combinations at 25 °C for 12 h. The reaction products were subjected to TLC and detected by staining with 4.5% TBA. Presence and absence of each enzyme is indicated with ‘+’ and ‘−’, respectively. Detailed conditions are shown under Section 4.

In the present study, we identified *flkin* and *flald* in the genome of strain UMI-01 as KDG kinase and KDPG aldolase gene, respectively. Since alginate lyases and DEH reductase in this strain have already been characterized [26,27,28], here we examined if the sequential action of these alginate-degrading and -assimilating enzymes could convert alginate to pyruvate and GAP in vitro. Namely, recombinant alginate lyases (recFlAlyA, recFlAlyB, and recFlAlex) [26,27], DEH reductase (recFlRed) [28], KDG kinase (recFlKin), and KDPG aldolase (recFlAld) were allowed to react alginate in various combinations, and each reaction product was analyzed by TLC (Figure 8) and quantified by thiobarbituric acid (TBA) and LDH–NADH methods (Table 2). As shown in Figure 8, alginate was almost completely degraded to DEH by the simultaneous actions of recFlAlyA, recFlAlyB, and recFlAlex. The DEH was also almost completely reduced to KDG by recFlRed. Furthermore, a major part of the KDG was converted to KDPG by recFlKin, and the band of KDPG became faint by the reaction of recFlAld. This indicated the splitting of KDPG to pyruvate and GAP by the action of recFlAld. Accordingly, the sequential action of recombinant enzymes was considered to be capable of converting alginate to pyruvate and GAP in vitro. Then, the yields of intermediates in each reaction step were quantified by TBA and LDH–NADH methods (Table 2). Concentrations of the unsaturated oligo-alginates, DEH, KDG, KDPG, and pyruvate (and GAP), were determined to be 4.2 mM, 9.8 mM, 9.8 mM, 8.1 mM, and 3.8 mM, respectively. Since the initial concentration of alginate (0.2% (*w*/*v*)) corresponds to 10 mM monosaccharide, the yields of DEH and KDG were estimated to be ~100%, and the yields of KDPG and pyruvate were estimated to be ~80% and ~40%, respectively. These results indicated that high-value intermediates such as KDPG could be produced from alginate with fairly high efficiency by the recombinant enzymes of the strain UMI-01 in vitro.

## 3. Discussion

### 3.1. Alginate-Metabolizing Enzymes of Flavobacterium *sp.* Strain UMI-01

**Figure 9:**
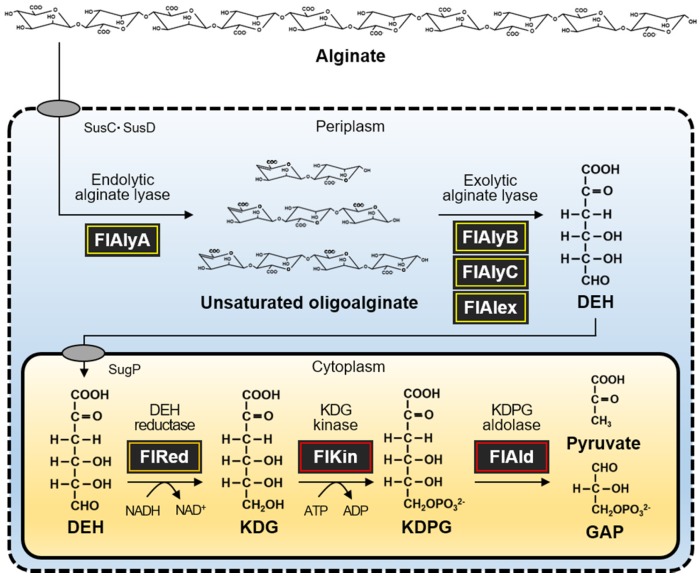
Alginate-metabolic system of *Flavobacterium* sp. strain UMI-01.

In the present study, *flkin* and *flald* in the genome of *Flavobacterium* sp. strain UMI-01 were confirmed to be the enzyme genes encoding KDG kinase and KDPG aldolase. The recombinant enzymes, recFlKin and recFlAld, showed KDG kinase and KDPG aldolase activity although low sequence identities were shown to the corresponding enzymes from other bacteria and archaea (Figure 2, Figure 3 and Figure 4). Consequently, these genes, along with previously reported alginate lyase and DEH reductase genes were confirmed to be the genes responsible for alginate metabolism of this bacterium. The alginate-metabolizing pathway of this strain is summarized as in Figure 9. The alginate lyases degrade polymer alginate to unsaturated monomer (DEH) in the periplasmic space [24,25]. DEH reductase, KDG kinase and KDPG aldolase convert DEH to pyruvate and GAP in the cytosol. Therefore, DEH produced in the periplasmic space should be incorporated to the cytosol by certain transportation system(s). Such DEH transporters in this strain have not been identified yet; however, sugar permease-like gene *sugp* and membrane transporter-like genes *susc* and *susd* were found in the operons A and B, respectively (see Figure 1). Thus, the putative permease and transporters are also indicated in Figure 9. Another problem is how the expressions of alginate-metabolizing genes are regulated. We recently noticed that expression levels of alginate lyases were significantly low in the absence of alginate but strongly increased by the addition of alginate to the medium. This indicates that the expressions of alginate-metabolic enzymes are up-regulated by alginate. We are now searching regulatory genes for alginate-metabolizing enzyme genes in the UMI-01 strain genome.

### 3.2. Properties of recFlKin and recFlAld

KDG kinase and KDPG aldolase are known to be the enzymes included in Entner–Doudoroff (ED) pathway. This pathway distributes over bacteria and archaea and play important roles in the metabolisms of glucuronate and glucose. In this pathway, KDG kinase phosphorylates KDG to KDPG, and KDPG aldolase split KDPG to pyruvate and GAP. Optimal temperature and pH of recFlKin were 50 °C and ~7.0, which were similar to those of KDG kinase from the bacteria *S. marcescens* [39]. While thermal stability of recFlKin was considerably low compared with the enzymes from archaea *S. tokodaii* [47] and *S. solfataricus* [40], e.g., these enzymes were stable up to 60–70 °C. recFlAld acts only on KDPG unlike archaea aldolases which split both KDG and KDPG [48,49]. Primary structures of bacterial aldolases showed low identity with those of archaea enzymes. The amino acid sequence of FlAld showed only 22%–25% identity with respect to *Proteobacteria* enzymes, while it showed 61%–65% identity with the *Bacteroidetes* enzymes. This suggests that somewhat deviated function between the *Proteobacteria* enzymes and *Bacteroidetes* enzymes. However, less different properties were found in recFlAld. Reverse reaction of bacterial aldolases was shown to be useful for the production of KDPG from pyruvate and GAP and also various compounds from pyruvate and aldehydes [44]. Our preliminary experiments also indicated that recFlAld could produce KDPG from pyruvate and GAP (data not shown, but see Section 2.3). Thus, recFlAld is also considered to be useful for producing novel compounds from pyruvate and various aldehydes.

### 3.3. Construction of In Vitro Alginate-Metabolizing System

An in vitro alginate-metabolizing system was successfully constructed from the recombinant enzymes, recFlAlyA, recFlAlyB, recFlAlex, recFlRed, recFlKin, and recFlAld. Accordingly, various kinds of intermediates could be produced by this system (Figure 8 and Table 2). Recently, alginate-assimilating enzymes of *S. degradans* 2-40^T^ were used for the production of KDG, KDPG, GAP and pyruvate [24,25]. However, the reaction efficiency of KDG kinase of *S. degradans* 2-40^T^ appeared to be lower than that of our system. Namely, the major part of KDG in the reaction mixture remained to be unphosphorylated in the *S. degradans* 2-40^T^ system. On the other hand, recFlKin in our system could convert KDG to KDPG with ~80% efficiency. This difference in the reaction efficiency between *S. degradans* enzyme and recFlKin may be derived from the origin of this enzyme, namely, from *Proteobacteria* species or *Bacteroidetes* species. To confirm this, we have to directly compare the KDG kinase properties between the enzymes from *Proteobacteria* and *Bacteroidetes* in future.

### 3.4. Production of a High-Value Product KDPG from Alginate

KDPG is a valuable leading compound for novel drugs and medicines. Synthesis of KDPG has been attempted by several methods [44,48,50]. For example, KDPG was first produced from gluconate with archaea enzymes [48]. However, this method required high-temperature reaction since the archaea enzymes are thermophilic. Reverse reaction of KDPG aldolase was also used for the production of KDPG from pyruvate and GAP [44,50]. However, this method required GAP, a significantly expensive raw material. On the other hand, we could produce KDPG from a much cheaper material, alginate, using the enzymes from the strain UMI-01. High recovery of KDPG from alginate (~80%) also indicated the practical potentiality of this enzyme. Thus, *Flavobacterium* sp. strain UMI-01 was considered to be a useful enzyme source for the production of value-added materials from alginate.

## 4. Experimental Section

### 4.1. Materials

Sodium alginate (*Macrocystis pyrifera* origin) was purchased from Sigma-Aldrich (St. Louis, MO, USA). Alginate-assimilating bacteria, *Flavobacterium* sp. strain UMI-01, was cultivated at 25 °C in a mineral salt (MS) medium including 1% (*w*/*v*) sodium alginate as described in our previous report [26]. Cell lysate (crude enzyme) of this strain was extracted from cell pellets by freeze and thaw followed by sonication as described previously [28]. DEH was prepared by the digestion of sodium alginate with the crude enzyme and purified by SuperQ-650S (Tosoh, Tokyo, Japan) anion-exchange chromatography [28]. Standard KDG, KDPG, pyruvate, and GAP were purchased from Sigma-Aldrich. pCold I expression vector was purchased from TaKaRa (Shiga, Japan) and modified to the form that can add 8 × Gly + 8 × His-tag to the C-terminus of the expressed proteins [26]. *E. coli* DH5α and BL21 (DE3) were purchased from TaKaRa. Ni-NTA resin was purchased from Qiagen (Hilden, Germany). A TLC silica gel 60 plate was purchased from Merk KGaA (Darmstadt, Germany). TSKgel DEAE-2SW (4.6 × 250 mm) and Superdex peptide 10/300 GL were purchased from Tosoh Bioscience LLC (King of Prussia, PA, USA) and GE Healthcare (Little Chalfont, Buckinghamshire, UK), respectively. Lactate dehydrogenase (LDH; porcine heart origin) and NADH were purchased from Oriental Yeast Co., LTD. (Tokyo, Japan). ATP and 9-aminoacridine were purchased from Sigma-Aldrich. Other chemicals were purchased from Wako Pure Chemical Industries Ltd. (Osaka, Japan).

### 4.2. Phylogenetic Analysis for KDG Kinases and KDPG Aldolases

Phylogenetic analysis was carried out using the amino acid sequences of KDG kinases or KDPG aldolases from *Proteobacteria, Bacteroidetes* and *Archaea* currently available. *Bacteroidetes* enzymes used are from *Gramella forsetii* KT0803, *Lacinutrix* sp. 5H-3-7-4, and *Dokdonia* sp. MED134, which were reported to be located in the alginolytic gene cluster of each species [51]. These amino acid sequences were first aligned with the sequences of FlKin or FlAld by the ClustalW program, then aligned sequences were trimmed with GBlocks. Phylogenetic trees were generated by the maximum likelihood algorithm on the basis of the LG model implemented in the Molecular Evolutionary Genetics Analysis version 6.0 (MEGA 6) software. The bootstrap values were calculated from 1000 replicates.

### 4.3. Cloning, Expression, and Purification of Recombinant FlKin and FlAld

Genomic DNA of strain UMI-01 was prepared with ISOHAIR DNA extraction kit (Nippon Gene, Tokyo, Japan). Coding regions of *flkin* and *flald,* 1023 bp and 669 bp, respectively, were amplified by PCR using specific primers including restriction sites, *Nco*I and *Bam*HI, in the 5′-terminal regions (Table 1). Genomic PCR was performed in a medium containing 10 ng of genomic DNA, 0.2 μM each primer, and Phusion DNA polymerase (New England Biolabs, Ipswich, MA, USA). The reaction medium was preincubated at 95 °C for 2 min, and a reaction cycle of 95 °C for 10 s, 55 °C for 20 s, and 72 °C for 60 s was repeated 30 times. The PCR product was ligated to pCold I vector pre-digested by *Nco*I and *Bam*HI using In-Fusion cloning system (Clontech Laboratories, Mountain View, CA, USA). Insertion of the genes in the vector was confirmed by nucleotide sequencing with DNA sequencer 3130xl (Applied Biosystems, Foster, CA, USA). Recombinant enzymes, recFlKin and recFlAld, were expressed with the pCold I–*E. coli* BL21 (DE3) system. The transformed BL21 (DE3) was inoculated to 500 mL of 2× YT medium and cultivated at 37 °C for 16 h. Then, the temperature was lowered to 15 °C and isopropyl β-D-1-thiogalactopyranoside was added to make the final concentration of 0.1 mM. After 24-h induction, bacterial cells were harvested by centrifugation at 5000× *g* for 5 min and suspended in a buffer containing 10 mM imidazole-HCl (pH 8.0), 0.5 M NaCl, 1% (*v*/*v*) TritonX-100, and 0.01 mg/mL lysozyme. The suspension was sonicated at 20 kHz (30W) for a total of 4 min (30 s × 8 times with each 1 min interval) and centrifuged at 10,000× *g* for 10 min. The supernatant containing recombinant proteins was mixed with 1 mL of Ni-NTA resin and incubated for 30 min on ice with occasional suspension. The resin was set on a disposal plastic column (1 × 5 cm) and washed three times with 20 mL of 30 mM imidazole-HCl (pH 8.0)–0.5 M NaCl. The recombinant proteins adsorbed to the resin were eluted with 250 mM imidazole-HCl (pH 8.0)–0.5 M NaCl and collected as 1 mL fractions. The fractions containing the recombinant proteins were pooled and dialyzed against 20 mM Tris-HCl (pH 7.4)–0.1 M NaCl.

### 4.4. Preparation of KDG

KDG was prepared from alginate using the crude enzyme of the strain UMI-01 as follows; 0.5% (*w*/*v*) sodium alginate (50 mL) was digested at 30 °C with 1 mg/mL of the crude enzyme, which contains alginate lyases and other metabolic enzymes. NADH was added to the mixture to make the final concentration 10 mM to reduce DEH with DEH reductase contained in the crude enzyme. After 12 h, four volumes of −20 °C 2-propanol were added to terminate the reaction and the proteins and NADH precipitated were removed by centrifugation at 10,000× *g* for 10 min. The supernatant containing KDG was dried up in a rotary evaporator at 35 °C. The dried powder was dissolved in 50 mL of distilled water and subjected to a TOYOPEARL SuperQ-650S column (2.4 × 22 cm) equilibrated with distilled water. The absorbed KDG and trace amount of unsaturated disaccharide were separately eluted by a linear gradient of 0–0.2 M NaCl in distilled water (total 400 mL). Elution of KDG and unsaturated disaccharide was detected by TBA reaction. In this chromatography, KDG was eluted at around 80 mM NaCl, while disaccharides were eluted at around 120 mM. Approximately 90 mg of KDG was obtained from 0.25 g of sodium alginate.

### 4.5. Preparation of KDPG

KDPG was prepared from the KDG by using recFlKin. Namely, recFlKin was (final concentration 10 μg/mL) was added to the reaction mixture (10 mL) containing 2.5 mM KDG, 2.5 mM ATP, 5 mM MgCl_2_, 20 mM Tris-HCl (pH 7.4), 100 mM KCl, and 1 mM dithiothreitol, and incubated at 40 °C for 3 h. The mixture was lyophilized, dissolved in 500 μL of distilled water and the supernatant was subjected to a Superdex peptide 10/300 GL column equilibrated with 0.1 M CH_3_COONH_4_. KDPG and KDG, which eluted together in this chromatography, were lyophilized, dissolved in 1 mL of distilled water, and subjected to HPLC (Shimadzu Prominence LC-6AD, Tokyo, Japan) equipped by TSKgel DEAE-2SW (Tosoh). KDG and KDPG were separately eluted at around 150 mM and 320 mM CH_3_COONH_4_ by the linear gradient of 0–0.4 M CH_3_COONH_4_. The amount of KDPG was quantified by the system comprising recFlAld and LDH–NADH using authentic KDPG as a standard. By the above procedure, 1.2 mg of KDPG was obtained from 4.5 mg of KDG.

### 4.6. Assay for KDPG Aldolase Activity

KDPG aldolase activity of recFlAld was assayed by the determination of pyruvate using a lactate dehydrogenase (LDH)–NADH coupling system [50]. Namely, the aldolase reaction was conducted at 30 °C in a reaction mixture containing 5 mM KDPG, 20 mM Tris-HCl (pH 7.4), 100 mM KCl, 1 mM DTT, and 1 μg/mL recFlAld in the presence of 0.2 mM NADH and 1 unit/mL LDH. The reaction rate was estimated from the decrease in the Abs 340 nm due to the oxidation of NADH accompanied by the reduction of pyruvate. One unit (U) of KDPG aldolase activity was defined as the amount of enzyme that produced 1 μmol of pyruvate per min.

### 4.7. Assay for KDG Kinase Activity

KDG kinase activity was assayed as follows. The reaction mixture containing 2.5 mM KDG, 2.5 mM ATP, 5 mM MgCl_2_, 20 mM Tris-HCl (pH 7.4), 100 mM KCl, 1 mM DTT, and 10 μg/mL recFlKin was incubated at 30 °C. At reaction times, 1, 15, and 30 min, an aliquot (160 μL) of the reaction mixture was taken out and heated at 100 °C for 3 min to terminate the reaction. To the mixture, 240 μL of a buffer containing 84 mM Tris-HCl (pH 7.4), 167 mM KCl, 0.67 mM NADH, 2.5 μg/mL recFlAld, and 1 unit of LDH was added and the pyruvate released was determined by the LDH–NADH system. One unit (U) of KDG kinase activity was defined as the amount of enzyme that produced 1 μmol of KDPG per min. Temperature dependence of recFlKin was determined at 10–60 °C. Thermal stability of recFlKin was assessed by measuring the activity remaining after the incubation at 10–50 °C for 30 min. pH dependence of recFlKin was determined with reaction mixtures adjusted to pH 4.5–5.3 with 20 mM CH_3_COONa buffer, pH 5.6–7.3 with 20 mM PIPES-NaOH buffer, pH 7.1–8.8 with 20 mM Tris-HCl buffer, and pH 9.1–9.7 with 20 mM glycine–NaOH buffer. The activity assay was conducted three times and the mean value was shown with standard deviation in each figure.

### 4.8. Construction of In Vitro Alginate-Metabolizing System from Recombinant Enzymes

An in vitro alginate-metabolizing system was constructed using recombinant alginate lyases (recFlAlyA, recFlAlyB, and recFlAlex) [26,27], recombinant DEH reductase (recFlRed) [28], and recFlKin and recFlAld prepared in the present study. Alginate-metabolizing reaction was conducted at 25 °C in a mixture containing 0.2% (*w*/*v*) sodium alginate, 10 mM NADH, 10 mM ATP, 10 mM MgCl_2_, 20 mM sodium phosphate (pH 7.4), 1 mM DTT, and various combinations of recFlAlyA, recFlAlyB, recFlAlex, recFlRed, recFlKin, and recFlAld with each final concentration at 10 μg/mL, 10 μg/mL, 10 μg/mL, 2.5 μg/mL, 10 μg/mL, and 1 μg/mL, respectively. After 12-h reaction, unsaturated oligo-alginates, DEH, and KDG, were analyzed by TLC and TBA reaction [52]. KDPG and pyruvate concentrations were determined by the LDH–NADH reaction.

### 4.9. Determination of Unsaturated Sugars

Unsaturated sugars were determined by the TBA method [52]. The sample containing unsaturated sugars (150 μL) was mixed with 150 μL of 20 mM NaIO_4_–0.125 M H_2_SO_4_ and allowed to react for 1 h on ice. Then, 100 μL of NaAsO_2_–0.5 N HCl was added to the mixture and incubated for 10 min at room temperature. To the mixture, 600 μL of 0.6% (*w*/*v*) TBA was added and heated for 10 min at 100 °C. The unsaturated sugars were determined by measuring Abs 548 nm, adopting the absorption coefficient for DEH and KDG, ε = 41 × 10^3^ M^−1^·cm^−1^, which we determined in the present study using KDG and DEH standards.

### 4.10. Thin-Layer Chromatography

TLC silica gel 60 plate was used for the analysis of the reaction products produced by recFlKin and recFlAld. The reaction product of recFlKin was prepared with a reaction mixture containing 2.5 mM KDG and 2.5 mM ATP and 200 μg/mL recFlKin. The reaction was carried out at 30 °C for 0–15 min and terminated by heating at 100 °C for 2 min. Four microliters of each reaction mixture was applied to a TLC plate. The reaction product was developed with 1-butanol:acetic acid:water = 2:1:1 (*v*:*v*:*v*) and detected by heating at 130 °C for 10 min after spraying 10% (*w*/*v*) sulfuric acid–90% (*w*/*v*) ethanol. The reaction product of recFlAld was prepared with a reaction mixture containing 5 mM KDPG and 1 μg/mL recFlAld. After the reaction at 30 °C for 0–15 min, six microliters of the reaction mixture were applied to TLC plate and developed with the same solvent as described above. The reaction product on the plate was detected with 0.5% (*w*/*v*) 2,5-dinitrophenylhydrazine (DNP)–20% (*v*/*v*) sulfuric acid–60% (*v*/*v*) ethanol. In case of unsaturated sugars, they were visualized with 4.5% (*w*/*v*) TBA after the periodic acid treatment.

### 4.11. Mass Spectrometry

Phosphorylation of KDG by recFlKin was detected by mass spectrometry. The KDG phosphorylated by recFlKin in the conditions described in Section 4.10 was mixed with 6.7 mg/mL 9-aminoacridine–methanol at 1:3 (*v*:*v*). One microliter of the mixture was applied to a sample plate and air-dried at room temperature. The sample was subjected to a matrix-assisted laser desorption ionization-time of flight mass spectrometer (MALDI-TOF-MS) (Proteomics Analyzer 4700, Applied Biosystems, Foster City, CA, USA) and analyzed in a negative-ion mode.

### 4.12. SDS-PAGE

SDS-PAGE was performed by the method of Porzio and Pearson [53] using 10% polyacrylamide gel. Proteins in the gel were stained with 0.1% (*w*/*v*) Coomassie Brilliant Blue R-250–50% (*v*/*v*) methanol–10% (*v*/*v*) acetic acid and the background of the gel was destained with 5% (*v*/*v*) methanol–7% (*v*/*v*) acetic acid.

### 4.13. Determination of Protein Concentration

Protein concentration was determined by the method of Lowry [54] using bovine serum albumin fraction V as a standard.

## 5. Conclusions

Enzymes responsible for the metabolism of alginate-derived DEH had not been well characterized in alginolytic bacteria. In the present study, KDG kinase-like gene *flkin* and KDPG aldolase-like gene *flald* in the genome of *Flavobacterium* sp. strain UMI-01 were investigated and the activities of the proteins encoded by these genes were assessed by using recombinant enzymes recFlKin and recFlAld. Analyses for reaction product of recFlKin and recFlAld indicated that these enzymes were KDG kinase and KDPG aldolase, respectively. Thus, the alginate metabolism of *Flavobacterium* sp. strain UMI-01 was considered to be achieved by the actions of FlKin and FlAld along with alginate lyases FlAlyA, FlAlyB and FlAlex, and DEH reductase FlRed. An in vitro alginate-metabolizing system was successfully constructed from the above enzymes. This system could convert alginate to pyruvate and GAP with 38% efficiency. This result indicates that the UMI-01 enzymes are available for the production of high-value materials like KDPG from alginate.

## Figures and Tables

**Table 1:** Primers used for amplification of *flkin* and *flald* genes.

Primer Name	Nucleotide Sequence
recFlKin-F	5′-AGGTAATACACCATGAAAAAAGTAGTCACTTTTGG-3′
recFlKin-R	5′-CACCTCCACCGGATCCTCTTGAAACTTTTCCTGAAA-3′
recFlAld-F	5′-ATGTAATACACCATGGCTCAATTTTCAAGAATAGA-3′
recFlAld-R	5′-CACCTCCACCGGATCCTTGTTTTAACTCTTTAATGA-3′

**Table 2:** Quantification of reaction products produced by the recombinant enzymes.

Enzymes	Substrates/Products	Concentration (mM)	Yield (%)
None	Alginate ^a^	10 ^a^	-
recFlAlyA	Oligoalginates	4.2 ± 0.06	-
recFlAlyA + recFlAlyB + recFlAlex	DEH	9.8 ± 0.34	98
recFlAlyA + recFlAlyB + recFlAlex + recFlRed	KDG	9.8 ± 1.0	98
recFlAlyA + recFlAlyB + recFlAlex + recFlRed + recFlKin	KDPG	8.1 ± 0.54	81
recFlAlyA + recFlAlyB + recFlAlex + recFlRed + recFlKin + recFlAld	Pyruvate (and GAP)	3.8 ± 0.33	38

^a^ 0.2% (*w*/*v*) sodium alginate theoretically corresponds to 10 mM monosaccharide.

## References

[1] Haug A., Larsen B., Smidsrod O. (1967). Studies on the sequence of uronic acid residues in alginic acid. Acta Chem. Scand..

[2] Gacesa P. (1988). Alginates. Carbohydr. Polym..

[3] Wong T.Y., Preston L.A., Schiller N.L. (2000). Alginate lyase: Review of major sources and enzyme characteristics, structure-function analysis, biological roles, and applications. Annu. Rev. Microbiol..

[4] Tomoda Y., Umemura K., Adachi T. (1994). Promotion of barley root elongation under hypoxic conditions by alginate lyase-lysate (A.L.L.). Biosci. Biotechnol. Biochem..

[5] Xu X., Iwamoto Y., Kitamura Y., Oda T., Muramatsu T. (2003). Root growth-promoting activity of unsaturated oligomeric uronates from alginate on carrot and rice plants. Biosci. Biotechnol. Biochem..

[6] Akiyama H., Endo T., Nakakita R., Murata K., Yonemoto Y., Okayama K. (1992). Effect of depolymerized alginates on the growth of bifidobacteria. Biosci. Biotechnol. Biochem..

[7] Ariyo B., Tamerler C., Bucke C., Keshavarz T. (1998). Enhanced penicillin production by oligosaccharides from batch cultures of *Penicillium chrysogenum* in stirred-tank reactors. FEMS Microbiol. Lett..

[8] Trommer H., Neubert R.H.H. (2005). Screening for new antioxidative compounds for topical administration using skin lipid model systems. J. Pharm. Pharm. Sci..

[9] Khodagholi F., Eftekharzadeh B., Yazdanparast R. (2008). A new artificial chaperone for protein refolding: Sequential use of detergent and alginate. Protein J..

[10] Mo S.-J., Son E.-W., Rhee D.-K., Pyo S. (2003). Modulation of tnf-α-induced icam-1 expression, no and h202 production by alginate, allicin and ascorbic acid in human endothelial cells. Arch. Pharm. Res..

[11] An Q.D., Zhang G.L., Wu H.T., Zhang Z.C., Zheng G.S., Luan L., Murata Y., Li X. (2009). Alginate-deriving oligosaccharide production by alginase from newly isolated *Flavobacterium* sp. LXA and its potential application in protection against pathogens. J. Appl. Microbiol..

[12] Enquist-Newman M., Faust A.M.E., Bravo D.D., Santos C.N.S., Raisner R.M., Hanel A., Sarvahowman P., Le C., Regitsky D.D., Cooper S.R. (2014). Efficient ethanol production from brown macroalgae sugars by a synthetic yeast platform. Nature.

[13] Wargacki A.J., Leonard E., Win M.N., Regitsky D.D., Santos C.N.S., Kim P.B., Cooper S.R., Raisner R.M., Herman A., Sivitz A.B. (2012). An engineered microbial platform for direct biofuel production from brown macroalgae. Science.

[14] Takeda H., Yoneyama F., Kawai S., Hashimoto W., Murata K. (2011). Bioethanol production from marine biomass alginate by metabolically engineered bacteria. Energy Environ. Sci..

[15] Miyake H., Yamaki T., Nakamura T., Ishibashi H., Fukuiri Y., Sakuma A., Komatsu H., Anso T., Togashi K., Umetani H. (2006). Gluconate dehydratase. U.S. Patent.

[16] Yoon H.J., Hashimoto W., Miyake O., Okamoto M., Mikami B., Murata K. (2000). Overexpression in *Escherichia coli*, purification, and characterization of *Sphingomonas* sp. A1 alginate lyases. Protein Expr. Purif..

[17] Hashimoto W., Miyake O., Momma K., Kawai S., Murata K. (2000). Molecular identification of oligoalginate lyase of *Sphingomonas* sp. strain A1 as one of the enzymes required for complete depolymerization of alginate. J. Bacteriol..

[18] Takase R., Ochiai A., Mikami B., Hashimoto W., Murata K. (2010). Molecular identification of unsaturated uronate reductase prerequisite for alginate metabolism in *Sphingomonas* sp. A1. Biochim. Biophys. Acta Proteins Proteom..

[19] Takase R., Mikami B., Kawai S., Murata K., Hashimoto W. (2014). Structure-based conversion of the coenzyme requirement of a short-chain dehydrogenase/reductase involved in bacterial alginate metabolism. J. Biol. Chem..

[20] Preiss J., Ashwell G. (1962). Alginic acid metabolism in bacteria I. J. Biol. Chem..

[21] Preiss J., Ashwell G. (1962). Alginic acid metabolism in bacteria II. J. Biol. Chem..

[22] Kim H.T., Ko H.J., Kim N., Kim D., Lee D., Choi I.G., Woo H.C., Kim M.D., Kim K.H. (2012). Characterization of a recombinant endo-type alginate lyase (Alg7D) from *Saccharophagus degradans*. Biotechnol. Lett..

[23] Kim H.T., Chung J.H., Wang D., Lee J., Woo H.C., Choi I.G., Kim K.H. (2012). Depolymerization of alginate into a monomeric sugar acid using Alg17C, an exo-oligoalginate lyase cloned from *Saccharophagus degradans* 2-40. Appl. Microbiol. Biotechnol..

[24] Takagi T., Morisaka H., Aburaya S., Tatsukami Y., Kuroda K., Ueda M. (2016). Putative alginate assimilation process of the marine bacterium *Saccharophagus degradans* 2-40 based on quantitative proteomic analysis. Mar. Biotechnol..

[25] Kim D.H., Wang D., Yun E.J., Kim S., Kim S.R., Kim K.H. (2016). Validation of the metabolic pathway of the alginate-derived monomer in *Saccharophagus degradans* 2-40^T^ by gas chromatography–mass spectrometry. Process Biochem..

[26] Inoue A., Takadono K., Nishiyama R., Tajima K., Kobayashi T., Ojima T. (2014). Characterization of an alginate lyase, FlAlyA, from *Flavobacterium* sp. strain UMI-01 and its expression in *Escherichia coli*. Mar. Drugs.

[27] Inoue A., Nishiyama R., Ojima T. (2016). The alginate lyases FlAlyA, FlAlyB, FlAlyC, and FlAlex from *Flavobacterium* sp. UMI-01 have distinct roles in the complete degradation of alginate. Algal Res..

[28] Inoue A., Nishiyama R., Mochizuki S., Ojima T. (2015). Identification of a 4-deoxy-l-erythro-5-hexoseulose uronic acid reductase, FlRed, in an alginolytic bacterium *Flavobacterium* sp. strain UMI-01. Mar. Drugs.

[29] Badur A.H., Jagtap S.S., Yalamanchili G., Lee J.-K., Zhao H., Rao C.V. (2015). Alginate lyases from alginate-degrading *Vibrio splendidus* 12B01 are endolytic. Appl. Environ. Microbiol..

[30] Shimizu E., Ojima T., Nishita K. (2003). cDNA cloning of an alginate lyase from abalone, *Haliotis discus hannai*. Carbohydr. Res..

[31] Suzuki H., Suzuki K.I., Inoue A., Ojima T. (2006). A novel oligoalginate lyase from abalone, *Haliotis discus hannai*, that releases disaccharide from alginate polymer in an exolytic manner. Carbohydr. Res..

[32] Rahman M.M., Inoue A., Tanaka H., Ojima T. (2010). Isolation and characterization of two alginate lyase isozymes, AkAly28 and AkAly33, from the common sea hare *Aplysia kurodai*. Comp. Biochem. Physiol. B Biochem. Mol. Biol..

[33] Rahman M.M., Inoue A., Tanaka H., Ojima T. (2011). cDNA cloning of an alginate lyase from a marine gastropod *Aplysia kurodai* and assessment of catalutically important residues of this enzyme. Biochimie.

[34] Inoue A., Mashino C., Uji T., Saga N., Mikami K., Olima T. (2015). Characterization of an eukaryotic PL-7 alginate lyase in the marine red alga *Pyropia yezoensis*. Curr. Biotechnol..

[35] Gacesa P. (1992). Enzymic degradation of alginates. Int. J. Biochem..

[36] Hobbs J.K., Lee S.M., Robb M., Hof F., Bar C., Abe K.T., Hehemann J.H., McLean R., Abbott D.W., Boraston A.B. (2016). KdgF, the missing link in the microbial metabolism of uronate sugars from pectin and alginate. Proc. Natl. Acad. Sci. USA.

[37] Mochizuki S., Nishiyama R., Inoue A., Ojima T. (2015). A novel aldo-keto reductase HdRed from the pacific abalone *Haliotis discus hannai*, which reduces alginate-derived 4-deoxy-l-erythro-5-hexoseulose uronic acid to 2-keto-3-deoxy-d-gluconate. J. Biol. Chem..

[38] Cynkin M.A., Ashwell G. (1960). Uronic acid metabolism in bacteria. J. Biol. Chem..

[39] Lee Y.S., Park I.H., Yoo J.S., Kim H.S., Chung S.Y., Chandra M.R.G., Choi Y.L. (2009). Gene expression and characterization of 2-keto-3-deoxy-gluconate kinase, a key enzyme in the modified Entner-Doudoroff pathway of *Serratia marcescens* KCTC 2172. Electron. J. Biotechnol..

[40] Kim S., Lee S.B. (2006). Characterization of *Sulfolobus solfataricus* 2-keto-3-deoxy-d-gluconate kinase in the modified Entner-Doudoroff pathway. Biosci. Biotechnol. Biochem..

[41] Lamble H.J., Theodossis A., Milburn C.C., Taylor G.L., Bull S.D., Hough D.W., Danson M.J. (2005). Promiscuity in the part-phosphorylative Entner-Doudoroff pathway of the archaeon *Sulfolobus solfataricus*. FEBS Lett..

[42] Potter J.A., Kerou M., Lamble H.J., Bull S.D., Hough D.W., Danson M.J., Taylor G.L. (2008). The structure of *Sulfolobus solfataricus* 2-keto-3-deoxygluconate kinase. Acta Crystallogr. Sect. D Biol. Crystallogr..

[43] Ohshima N., Inagaki E., Yasuike K., Takio K., Tahirov T.H. (2004). Structure of *Thermus thermophilus* 2-keto-3-deoxygluconate kinase: Evidence for recognition of an open chain substrate. J. Mol. Biol..

[44] Shelton M.C., Cotterill I.C., Novak S.T.A., Poonawala R.M., Sudarshan S., Toone E.J. (1996). 2-Keto-3-deoxy-6-phosphogluconate aldolases as catalysts for stereocontrolled carbon-carbon bond formation. J. Am. Chem. Soc..

[45] Allard J., Grochulski P., Sygusch J. (2001). Covalent intermediate trapped in 2-keto-3-deoxy-6-phosphogluconate (KDPG) aldolase structure at 1.95-A resolution. Proc. Natl. Acad. Sci. USA.

[46] Bell B.J., Watanabe L., Lebioda L., Arni R.K. (2003). Structure of 2-keto-3-deoxy-6-phosphogluconate (KDPG) aldolase from *Pseudomonas putida*. Acta Crystallogr. D Biol. Crystallogr..

[47] Ohshima T., Kawakami R., Kanai Y., Goda S., Sakuraba H. (2007). Gene expression and characterization of 2-keto-3-deoxygluconate kinase, a key enzyme in the modified Entner-Doudoroff pathway of the aerobic and acidophilic hyperthermophile *Sulfolobus tokodaii*. Protein Expr. Purif..

[48] Ahmed H., Ettema T.J.G., Tjaden B., Geerling A.C.M., Oost J.V.D., Siebers B. (2005). The semi-phosphorylative Entner-Doudoroff pathway in hyperthermophilic archaea: A re-evaluation. Biochem. J..

[49] Reher M., Fuhrer T., Bott M., Schönheit P. (2010). The nonphosphorylative entner-doudoroff pathway in the thermoacidophilic euryarchaeon *Picrophilus torridus* involves a novel 2-Keto-3-deoxygluconate-specific aldolase. J. Bacteriol..

[50] Cotterill I.C., Shelton M.C., Machemer D.E.W., Henderson D.P., Toone E.J. (1998). Effect of phosphorylation on the reaction rate of unnatural electrophiles with 2-keto-3-deoxy-6-phosphogluconate aldolase. J. Chem. Soc. Trans..

[51] Kabisch A., Otto A., Konig S., Becher D., Albrecht D., Schuler M., Teeling H., Amann R.I., Scheweder T. (2014). Functional characterization of polysaccharide utilization loci in the marine *Bacteroidetes* ‘*Gramella forsetii*’ KT0803. ISME J..

[52] Weissbach A., Hurwitz J. (1959). The Formation of 2-Keto-3-deoxyheptonie Acid in Extracts of *Escherichia coli* B. I. Identification. J. Biol. Chem..

[53] Porzio M.A., Pearson A.M. (1977). Improved resolution of myofibrillar proteins with sodium dodecyl sulfate-polyacrylamide gel electrophoresis. Biochim. Biophys. Acta (BBA) Protein Struct..

[54] Lowry O.H., Rosebrough N.J., Farr A.L., Randall R.J. (1951). Protein measurement with the dolin phenol reagent. J. Biol. Chem..

